# Rotating Hybrid Nanofluid Flow with Chemical Reaction and Thermal Radiation between Parallel Plates

**DOI:** 10.3390/nano12234177

**Published:** 2022-11-24

**Authors:** Mubashar Arshad, Ali Hassan, Qusain Haider, Fahad M. Alharbi, Najah Alsubaie, Abdullah Alhushaybari, Diana-Petronela Burduhos-Nergis, Ahmed M. Galal

**Affiliations:** 1 Department of Mathematics, University of Gujrat, Gujrat 50700, Pakistan; 2 Department of Mathematics, Al-Qunfudah University College, Umm Al-Qura University, Mecca, Saudi Arabia; 3 Department of Computer Sciences, College of Computer and Information Sciences, Princess Nourah bint Abdulrahman University, P.O. Box 84428, Riyadh 11671, Saudi Arabia; 4 Department of Mathematics, College of Science, Taif University, P.O. Box 11099, Taif 21944, Saudi Arabia; 5 Faculty of Materials Science and Engineering, “Gheorghe Asachi” Technical University, 700050 Iasi, Romania; 6 Department of Mechanical Engineering, College of Engineering in Wadi Alddawasir, Prince Sattam bin Abdulaziz University, Saudi Arabia; 7 Production Engineering and Mechanical Design Department, Faculty of Engineering, Mansoura University, Mansoura P.O. Box 35516, Egypt

**Keywords:** rotational system, porous surface, hybrid nanofluid, heat source/sink, magneto hydrodynamic, radiation, chemical reaction

## Abstract

This research investigates the two different hybrid nanofluid flows between two parallel plates placed at two different heights, $y_{0}\;$and $y_{h}$, respectively. Water-based hybrid nanofluids are obtained by using $Al_{2}O_{3}$, $TiO_{2}$ and Cu as nanoparticles, respectively. The upper-level plate is fixed, while the lower-level plate is stretchable. The fluid rotates along the *y*-axis. The governing equations of momentum, energy and concentration are transformed into partial differential equations by using similarity transformations. These transformed equations are grasped numerically at MATLAB by using the boundary value problem technique. The influence of different parameters are presented through graphs. The numerical outcomes for rotation, Nusselt, Prandtl, and Schmidt numbers are obtained in the form of tables. The heat transfer rate increases by augmentation in the thermophoresis parameter, while it decays by increasing the Reynolds number. Oxide nanoparticles hybrid nanofluid proved more efficient as compared to mixed nanoparticles hybrid nanofluid. This research suggests using oxide nanoparticles for good heat transfer.

## 1. Introduction

A crucial component of numerous industrial and technological processes is the fluid flow above a stretched surface with mass and heat transfer. Applications include the chilling of sheets, melt-spinning operations, fiber spinning, casting, etc. Additionally, fluids flow is used in geophysics, industrial engineering, petroleum engineering, groundwater hydrology, ceramic engineering, and chemical engineering. The final finishing of products depends upon the amount of heat transfer. Therefore, it is crucial to check the velocity and heat transfer inside a fluid to obtain a basic understanding of these processes.

Crane [1] gave the concept of boundary layer flow for viscous incompressible fluid over a stretchable surface. Using this concept, researchers have shown their interest in boundary layer flows during the last few decades. Dutta et al. [2] looked at the heat transfer in the flow by a stretchable surface with uniform heat source. A computational investigation for MHD flow in a rectangular cavity discussed by Hassan et al. [3]. Exponentially extending surfaces were used by Nadeem and Lee [4] to characterize the boundary layer flow.

Non-Newtonian nanofluids do not obey the Newton law of viscosity, while Newtonian nanofluids obey this law because they have direct relations between shear stress and shear rate when subjected to applied stress. Such fluid plays an essential part in different industrial applications such as polymer extrusion, cosmetics, coolants, in the food industry and the final finishing of the products. The nanofluids are prepared by suspending nanometer-sized particles in a host fluid, and these fluids have improved heat transfer rates as compared to the host fluid. Similarly, hybrid nanofluids are the mixture of one or more nanoparticles in nanofluids that perform extraordinary heat transmission. Sarada et al. [5] considered the non-Newtonian fluid flow above the stretching surface with variable thermal conditions. Punith et al. [6] explored the three-dimensional non-Newtonian magnetic fluid flow over the stretchable surface. Wong and De Leon [7] described the more detailed applications for nanofluids. Additionally, Punith et al. [8] explored the non-Newtonian nanofluid flow with chemical reaction and activation energy. Numerical solution for non-Newtonian nanofluid presented by Nadeem et al. [9]. For the transport of nanofluid through a stretching surface, Ghasemi et al. [10] used spectrum relaxation approach. Arshad et al. [11] used the porous stretching surface to examine the fluid considering the chemical reaction.

Rout and Mishra [12] gave a comparative study for the magnetohydrodynamic nanofluid flow above extending surface and proved that a higher radiation rate increases the heat transfer rate. Reddy et al. [13] gave the unsteady MHD nanofluid flow above a slandering stretching sheet with a slip effect. The power law index determines whether a fluid is non-Newtonian or Newtonian and with the help of this law, Raju et al. [14] described a dual solution for 3D magnetohydrodynamic nanofluid flow over a permeable stretching surface. Umavathi et al. [15] investigated the MHD flow between parallel convectively heated disks for Casson nanofluid. Hussain et al. [16] explored the time-dependent flow to analyze the heat transmission in cylinder. Naveen et al. [17] utilized the KKL model to explore the magnetic dipole effect on radiative fluid flow. Ziaei-Rad et al. [18] elaborated on the dissipation of MHD nanofluid flow with an artificial neural network technique.

Many industrial uses involve different types of heat flow like in plastic industries, molding, blowing, and extrusion of plastic. Similarly, converting industries involve presses, rolls, laminating, and printing. Nuclear power plants involve radiations that play an important role. Zeeshan et al. [19] explored the magnetic dipole influence on viscous fluid with thermal radiation. Muhammad et al. [20] used carbon nanotubes for the investigation of MHD flow with heat source/sink and thermal radiation. Jamshed et al. [21] used the second grade nanofluid to discuss the radiative heat transfer flow. Prasannakumara and Gowda [22] investigated the heat and mass transfer for radiative heat flux with a uniform magnetic field. Soumya et al. [23] involved the non-linear radiation and slip conditions to explore the kerosene and water-based hybrid nanoparticles nanofluid in the suction/injection process. Jayaprakash et al. [24] recently elaborated on the convective heat transfer and activation energy performance in radiative hybrid nanofluid flow. Hussain et al. [25] explored the hybrid nanofluid flow for single-wall carbon nanotubes and multi-walls carbon nanotubes along the thermal radiation. Ramesh and Gireesha [26] investigated the dusty fluid flow with radiation above the stretching surface. Hussain et al. [27] computationally explored the MHD three-dimensional nanofluid flow above the stretchable surface with linear and non-linear radiation.

The thermophoresis phenomenon is defined as the particle’s migration from a higher temperature to a lower temperature. This technique is very useful for the accumulation of particles like the movement of holes of electrical charge in semiconductors. Tsai et al. [28] presented the thermophoretic decomposition of particles in steady state and unsteady flows. Recently, Arshad et al. [29] presented the Brownian motion and thermophoresis effect over the non-linear stretchable surface. Hassan et al. [30] considered the hybrid nanoparticles nanofluid under radiation for heat transportation analysis. Qin et al. [31] analyzed the thermal and solutal transport of Blasius–Rayleigh–Stokes flow with convective boundary conditions for hybrid nanofluid flow. Madhukesh et al. [32] presented the influence of thermophoretic particle migration in the flow of a hybrid nanofluid above a thin turning needle. Ullah et al. [33] explored the unsteady stretchable surface with thermophoresis and Brownian motion effect for Reiner-Philippoff fluid. Sensitivity testing and numerical analysis of the tangent hyperbolic nanofluid flow on a stretching surface is done by Shafiq et al. [34]. Khan et al. [35] used the convective boundary conditions to elaborate the heat and mass transfer in 3D flow over a stretchable surface. Arshad et al. [36] explored the MHD flow over an exponentially stretching surface with thermophoresis and Brownian motion. Pal et al. [37] investigated the thermophoresis and Brownian motion influence on magneto-convective heat transfer above the stretching surface for viscoelastic fluid.

The temperature gradient is a physical quantity, which describes at which rate and in which direction temperature is being transferred around a particular region. Similarly, the concentration gradient is defined as the mass transfer rate. These types of flow have a crucial impact on various applications. Makinde et al. [38] presented the boundary layer flow for exponentially stretching surface with heat sink/source and thermal radiation Hamid et al. [39] investigated the bio-convection flow of magneto-cross nanofluid including microorganisms by using an effective Prandtl number technique. Varun Kumar et al. [40] explored the Arrhenius activation energy for hybrid nanofluid fluid above a curved stretching surface. Shah et al. [41] used the Prandtl hybrid nanofluid flow with chemical reactions and motile microorganisms to study the bio-convection effects. Faraz et al. [42] explored the multi-slip effect on axisymmetric Casson fluid flow with a chemical reaction. Arshad and Hassan [43] numerically investigated the hybrid nanofluid flow over permeable stretching surface considering magnetic field. Hassan et al. [44] explored viscous dissipation and heat absorption with chemical reactions and heat source/sink. Krishnamurthy et al. [45] explored the chemical reaction effects and melting heat transfer for frontier layer slip flow.

The main objective of this research is to examine the flow of a viscous, incompressible, hybrid nanofluid above a stretchy, rotating, permeable plate with a heat source and chemical reaction under the influence of a magnetic field. The fluid and plates are rotating simultaneously with constant speed about the axis of rotation. The governing equations of momentum, energy and concentration are transformed into ODEs by a similarity transformation and tackled at MATLAB using the boundary value problem technique. The influence of different constraints is discussed in the form of graphs and tables.

## 2. Problem Formulation

Suppose a rotating three-dimensional, steady, incompressible, and electrically conducting hybrid nanofluid flow between two permeable parallel plates. The cartesian coordinate system is considered to understand the problem as the *y*-axis is perpendicular to the *x*-axis and the *z*-axis is perpendicular to both axes. The lower level is placed at a height $y_{0}$ and upper-level plate at a height $y_{h}.$ The lower-level plate is being stretched with rate U=ax, i.e., proportional to the applied equal and opposite forces maintaining the origin (0,0,0) of plates fixed. A uniform magnetic field $B_{0}$ is applied parallel to *y*-axis in which fluid is rotating. Two different namely $Al_{2}O_{3}/TiO_{2}$-water and $Al_{2}O_{3}/Cu$-water hybrid nanofluids are considered for comparison. The heat sink/source, chemical reaction, mixed convection, thermophoresis, and Brownian motion are considered to investigate the effects on different profiles. The governing equations have the following form:

**Continuity Equation:**

(1) $$\frac{\partial u}{\partial x}\;+\;\frac{\partial v}{\partial y}+\;\frac{\partial w}{\partial z}\;\;\;=0,$$

**Momentum Equations:**

(2) $$\rho_{hnf}\left(u\frac{\partial u}{\partial x}\;+\;v\;\frac{\partial u}{\partial y}+2\Omega w\right)=\frac{\partial p^{*}}{\partial x}+\mu_{hnf}\left(\frac{\partial^{2}u}{\partial x^{2}}+\frac{\partial^{2}u}{\partial y^{2}}+\frac{\partial^{2}u}{\partial z^{2}}\right)-\sigma_{hnf}B_{0}u-\frac{\mu_{hnf}}{k_{o}}u+g^{*}{\left(\rho \beta_{T}\right)}_{hnf}\left(T-T_{\infty }\right),$$

(3) $$\rho_{hnf}\left(v\frac{\partial v}{\partial y}\right)=-\frac{\partial p^{*}}{\partial y}+\mu_{hnf}\left(\frac{\partial^{2}v}{\partial x^{2}}+\frac{\partial^{2}v}{\partial y^{2}}+\frac{\partial^{2}v}{\partial z^{2}}\right),$$

(4) $$\rho_{hnf}\left(u\frac{\partial w}{\partial x}\;+\;v\;\frac{\partial w}{\partial y}-2\Omega v\right)=\mu_{hnf}\left(\frac{\partial^{2}w}{\partial x^{2}}+\frac{\partial^{2}w}{\partial y^{2}}\right)-\sigma_{hnf}B_{0}w-\frac{\mu_{hnf}}{k_{o}}w,$$

**Energy Equation:**

(5) $$\begin{array}{cc} u\frac{\partial T}{\partial x}\;+\;v\;\frac{\partial T}{\partial y} & +w\frac{\partial T}{\partial z}\\ & =\alpha \left(\;\frac{\partial^{2}T}{\partial x^{2}}+\frac{\partial^{2}T}{\partial y^{2}}+\frac{\partial^{2}T}{\partial z^{2}}\right)\\ & +\frac{{\left(\rho C_{p}\right)}_{s}}{{\left(\rho C_{p}\right)}_{f}}\left[D_{B}\left\{\frac{\partial C}{\partial x}.\frac{\partial T}{\partial x}+\frac{\partial C}{\partial y}.\frac{\partial T}{\partial y}+\frac{\partial C}{\partial z}.\frac{\partial T}{\partial z}\right\}+\frac{D_{T}}{T_{c}}\left\{{\left(\frac{\partial T}{\partial x}\right)}^{2}+{\left(\frac{\partial T}{\partial y}\right)}^{2}+{\left(\frac{\partial T}{\partial z}\right)}^{2}\right\}\right]-\frac{1}{{\left(\rho C_{p}\right)}_{hnf}}\frac{\partial q_{r}}{\partial y}\\ & +\frac{Q_{o}}{{\left(\rho C_{p}\right)}_{hnf}}\left(T-T_{\infty }\right) \end{array}$$

**Concentration Equation:**

(6) $$u\frac{\partial C}{\partial x}\;+\;v\;\frac{\partial C}{\partial y}+w\frac{\partial C}{\partial z}=D_{B}\left(\frac{\partial^{2}C}{\partial x^{2}}+\frac{\partial^{2}C}{\partial y^{2}}+\frac{\partial^{2}C}{\partial z^{2}}\right)+\frac{D_{T}}{T_{0}}\left\{\frac{\partial^{2}T}{\partial x^{2}}\times \frac{\partial^{2}T}{\partial y^{2}}\times \frac{\partial^{2}T}{\partial z^{2}}\right\}-\frac{K}{{\left(\rho C_{p}\right)}_{hnf}}\left(C-C_{\infty }\right)$$

Here, u,v and w are velocity components in x,y and z directions, respectively, the parameters $T,\;C,K,\;Q_{0}\;k_{0},\;D_{B},\;q_{r}$ are all defined in the nomenclature section. The missing term $\frac{\partial p^{*}}{\partial z}\;$in Equation (4) shows that there is a net cross-flow along the *z*-axis The Rosseland thermal radiation approximation relation is defined as $q_{r}=-\frac{4\sigma^{*}}{3k^{*}}\frac{\partial T^{4}}{\partial y}.$ Where $\sigma^{*}$. describes the Stefan–Boltzmann constant, and $k^{*}$ presents absorption coefficient. The temperature difference is assumed to be very small, and Taylor’s series expansion of $T^{4}$ in terms of $T_{\infty }$ will truncate the temperature to $T^{4}\approx 4TT_{\infty }{}^{3}-3T_{\infty }{}^{3}$. Thus, using this truncated form will provide us with the following simplified form:

(7) $$\frac{\partial q_{r}}{\partial y}=-\frac{16\sigma_{1}T_{\infty }{}^{3}}{3k_{1}}\frac{\partial^{2}T}{\partial y^{2}},$$

The corresponding boundary conditions are:

(8) $$\begin{array}{c} u=ax,\;v=0,\;w=0,\;T=T_{h},\;C=C_{h}\;\;\;\;\;\;\;\;\;\;\;\;\;\;\;\;\;\;\;\;\;\;\;\;\;\;\;\;at\;y_{0}\;\;\\ u=0,\;v=v_{0},\;w=0,\;T=T_{0},\;C=C_{0}\;\;\;\;\;\;\;\;\;\;\;\;\;\;\;\;\;\;\;\;\;\;\;\;\;\;\;\;\;\;\;\;at\;\;y_{h}\;\;\; \end{array}\}$$

**Similarity Transformation:**

The following similarity transformation is utilized to convert leading equations into a set of ordinary differential equations.

(9) $$u=axf^{\prime }\left(\eta \right),\;\;v=-ahf\left(\eta \right),\;\;w=axg\left(\eta \right),\;\;\eta =\frac{y}{h}\;\theta \left(\eta \right)\left(T_{0}-T_{h}\right)=T-T_{h},\;\;\phi \left(\eta \right)\left(C_{0}-C_{h}\right)=C-C_{h}$$

The differentiation is with respect to η.

**Transformed Governing Equations:**

The flow governing equalities of continuity, momentum, energy and concentration takes the following form after employing the similarity transformation. The continuity equation is identically satisfied, and the remaining are:

(10) $$\frac{-1}{a^{2}x\rho_{hnf}}\frac{\partial p^{*}}{\partial x}=\frac{-f^{\prime \prime \prime }}{RH_{1}{\left(1-\varphi_{1}\right)}^{5/2}{\left(1-\varphi_{2}\right)}^{5/2}}+\frac{H_{4}}{H_{1}}\frac{M}{R}f^{\prime }+\left({f^{\prime }}^{2}-ff^{\prime \prime }\right)+\frac{2K_{r}g}{R}+Zf^{\prime }-\frac{\theta \gamma H_{5}}{H_{1}},$$

(11) $$\frac{-1}{a^{2}x\rho_{hnf}}\frac{\partial p^{*}}{\partial y}=ff^{\prime }-\frac{f^{\prime \prime }}{RH_{1}{\left(1-\varphi_{1}\right)}^{5/2}{\left(1-\varphi_{2}\right)}^{5/2}},$$

(12) $$\frac{g^{\prime \prime }}{{\left(1-\varphi_{1}\right)}^{5/2}{\left(1-\varphi_{2}\right)}^{5/2}}-RH_{1}\left(gf^{\prime }-fg^{\prime }\right)+2K_{r}H_{1}f^{\prime }-MgH_{4}-Zg=0.$$

By removing the pressure gradient term from above mentioned Equations (10) and (11) reduced to

(13) $$\frac{f^{\prime \prime \prime }}{RH_{1}{\left(1-\varphi_{1}\right)}^{5/2}{\left(1-\varphi_{2}\right)}^{5/2}}-\frac{H_{4}}{H_{1}}\frac{M}{R}f^{\prime }-\left({f^{\prime }}^{2}-ff^{\prime \prime }\right)-\frac{2K_{r}g}{R}-Zf^{\prime }+\frac{\theta \gamma H_{5}}{H_{1}}=H_{2}$$

Here, $H_{2}\;$is a constant. Afterwards, differentiation Equation (13), with respect to η, takes the form:

(14) $$f^{iv}-{\left(1-\varphi_{1}\right)}^{5/2}{\left(1-\varphi_{2}\right)}^{5/2}\left[H_{4}Mf^{\prime \prime }+RH_{1}\left(f^{\prime }f^{\prime \prime }-ff^{\prime \prime \prime }\right)+2K_{r}g^{\prime }H_{1}+H_{1}Zf^{\prime \prime }-\frac{\theta^{\prime }\gamma H_{5}}{H_{1}}\right]=0.$$

So, the leading equations of momentum/velocity of the current problem take the following form:

(15) $$f^{iv}-{\left(1-\varphi_{1}\right)}^{5/2}{\left(1-\varphi_{2}\right)}^{5/2}\left[H_{4}Mf^{\prime \prime }+RH_{1}\left(f^{\prime }f^{\prime \prime }-ff^{\prime \prime \prime }\right)+2K_{r}g^{\prime }H_{1}+H_{1}Zf^{\prime \prime }-\frac{\theta^{\prime }\gamma H_{5}}{H_{1}}\right]=0,$$

(16) $$g^{\prime \prime }-{\left(1-\varphi_{1}\right)}^{5/2}{\left(1-\varphi_{2}\right)}^{5/2}\left[RD_{1}\left(gf^{\prime }-fg^{\prime }\right)-2K_{r}D_{1}f^{\prime }+MgD_{4}+Zg\right]=0.$$

The Roseland approximation in the energy equation for radiative heat flux [19] is defined as:

(17) $$q_{r}=-\frac{4\sigma^{*}}{3k^{*}}\frac{\partial T^{4}}{\partial y}$$

Here, $\sigma^{*}\;$is he Stefan–Boltzman constant and $k^{*}\;$is the absorption coefficient. Taking into consideration the minimal difference of the temperature, the Taylor series expansion for $T^{4}$ in terms of$\;T_{\infty }$, can be written as follows:

(18) $$T^{4}\approx 4TT^{3}{}_{\infty }-3T^{3}{}_{\infty }$$

Energy equality takes the form by utilizing the definition of heat flux$\;q_{r}$:

(19) $$\theta^{\prime \prime }\left(1+\frac{1}{D_{3}}\frac{4}{3\;}Rd\right)+PrRf\theta^{\prime }+N_{b}\phi^{\prime }\theta^{\prime }+N_{t}{\theta^{\prime }}^{2}-\frac{Q\theta }{H_{3}}=0,$$

Similarly, the following form (dimensionless) takes the concentration Equation (6):

(20) $$\phi^{\prime \prime }+R.Sc.f.\phi^{\prime }+\frac{N_{t}}{N_{b}}\theta^{\prime \prime }+\phi \frac{H_{6}}{H_{3}}=0.$$

The transformed boundary conditions are:

(21) $$\begin{array}{c} f\left(0\right)=0,\;\;f^{\prime }\left(0\right)=1,\;\;g\left(0\right)=0,\;\;\theta =1,\;\;\phi =1\;\;\;\;\;\;at\;\;\;\;\eta =0\\ f\left(1\right)=\lambda ,\;\;f^{\prime }\left(1\right)=0,\;\;g\left(1\right)=0,\;\;\theta =0,\;\;\phi =0\;\;\;\;\;\;\;\;\;at\;\;\;\;\;\;\eta =1\;\;\;\; \end{array}\}$$

The $\lambda =\frac{v_{0}}{ah}$ is a dimensionless suction/injection parameter.

The dimensionless quantities magnetic field, porosity, mixed convection, Prandtl number, Schmidt number, Reynolds number, radiation, Brownian motion, thermophoresis, radiation, heat source/sink, chemical reaction and constants for hybrid nanofluid are defined below as:

(22) $$\begin{array}{c} M=\frac{\sigma_{f}B_{0}{}^{2}h^{2}}{\rho_{f}\mu_{f}},Z=\frac{\mu_{hnf}}{a\rho_{hnf}k_{o}},\gamma =\frac{Gr_{x}}{Re_{x}{}^{2}},\;Pr=\frac{\mu_{f}}{\alpha \;\rho_{f}},\;Sc=\frac{\mu_{f}}{D_{B}\;\rho_{f}},\\ K_{r}=\frac{\;\rho_{f}\Omega h^{2}}{\mu_{f}},\;R=\frac{\;\rho_{f}ah^{2}}{\mu_{f}},\;N_{b}=\frac{{\left(\rho C_{p}\right)}_{s}D_{B}C_{h}}{\alpha {\left(\rho C_{p}\right)}_{f}},\;N_{t}=\frac{{\left(\rho C_{p}\right)}_{s}D_{T}T_{h}}{\alpha {\left(\rho C_{p}\right)}_{f}T_{c}},\;\;Rd=\frac{4\sigma^{*}T^{3}{}_{\infty }}{k_{f}k^{*}},\\ Q=\frac{Q_{0}h^{2}}{k_{f}},\;K_{c}=Kh^{2},H_{1}=\frac{\rho_{hnf}}{\rho_{f}},H_{3}=\frac{k_{hnf}}{k_{f}}H_{4}=\frac{\sigma_{hnf}}{\sigma_{f}},\;H_{5}=\frac{\rho \beta_{Thnf}}{\rho \beta_{f}},\\ H_{6}=\frac{{\left(\rho C_{p}\right)}_{hnf}}{{\left(\rho C_{p}\right)}_{f}},\\ where\;Gr_{x}=\frac{g^{*}\beta_{Thnf}}{\nu_{f}{}^{2}}x^{3}\left(T-T_{\infty }\right)\;and\;Re_{x}=\frac{u\left(x\right)}{\nu_{f}} \end{array}\}$$

**Quantities of Physical Interest:**

The skin resistance coefficient Cf along the stretchable wall is the physical quantity of concern in this problem, this one is defined as

(23) $$Cf_{x}=\frac{\tau_{wx}}{\rho_{f}u_{w}{}^{2}},\;\;Cf_{z}=\frac{\tau_{wz}}{\rho_{f}u_{w}{}^{2}}$$

Here, $\tau_{w}$ indicates the shear tension or skin friction along the stretched wall, and is defined as

(24) $$\tau_{wx}=\mu_{hnf}{\left(\frac{\partial u}{\partial y}\right)}_{y=0},\;\;\tau_{wz}=\mu_{hnf}{\left(\frac{\partial w}{\partial y}\right)}_{y=0}$$

The dimensionless form is:

(25) $$\tilde{Cf_{x}}=\frac{\mu_{hnf}}{\mu_{f}}\;f^{\prime \prime }\left(0\right),\;\;\tilde{Cf_{z}}=\frac{\mu_{hnf}}{\mu_{f}}\;g^{\prime }\left(0\right),\;$$

Here $\tilde{Cf_{x}}=\frac{Rx}{h}Cf_{x}$ and$\tilde{\;Cf_{z}}=\frac{Rx}{h}Cf_{z}$.

The temperature field can be used to compute the Nusselt number (dimensionless) for the temperature transference constant at the surface:

(26) $$Nu_{x}=\frac{hq_{w}}{k_{f}\left(T_{0}-T_{h}\right)},\;\;q_{w}=-k_{hnf}{\left(\frac{\partial T}{\partial y}\right)}_{y=0}+{\left(q_{r}\right)}_{w},$$

or

(27) $$Nu_{x}=-\left(D_{3}+\frac{4}{3}Rd\right)\theta^{\prime }\left(0\right),$$

The concentration field can be used to compute the non-dimensional form of the mass transfer coefficient at the sheet in terms of the Sherwood number:

(28) $$Sh_{x}=\frac{hq_{m}}{D_{B}\left(C_{0}-C_{h}\right)},\;\;q_{m}=-D_{B}{\left(\frac{\partial C}{\partial y}\right)}_{y=0},\;\;Sh_{x}=-\phi^{\prime }\left(0\right),$$

## 3. Solution Methodology

**Figure 1:**
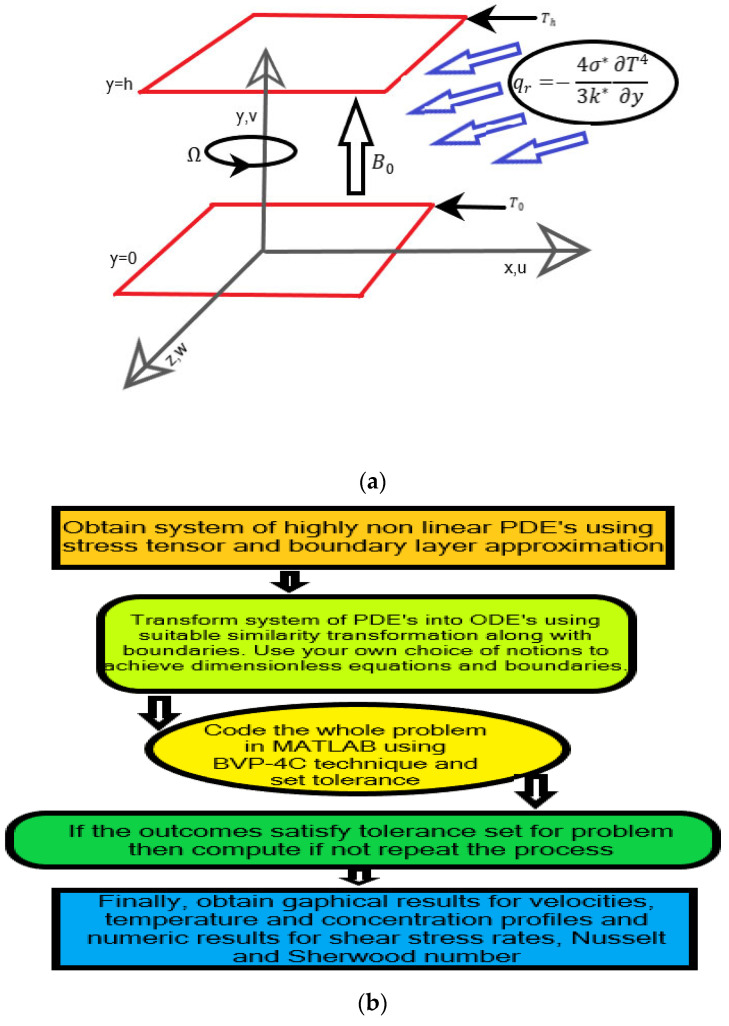
(**a**) The schematic diagram of the problem. (**b**) Algorithm of the solution.

Figure 1b shows the complete algorithm of the solution. The boundary value problem technique is used to tackle the system of nonlinear ODE1a Equations (15), (16), (19) and (20) with boundary conditions Equation (21) at MATLAB. The advantage of this technique is that it is easy to use, time-saving and provides accurate outcomes. The complete methodology provides insight into how to collect the data, data processing and analysis of the obtained outcomes. The following steps illustrate the complete methodology:

1. Obtain the highly non-linear system of PDEs using the boundary layer approximation (BLA) and stress tensor.
2. Converting the achieved PDEs into ODEs with the help of suitable similarity transforms.
3. Transforming a set of ODEs and associated boundaries into first-order ordinary differential equations so that we can easily call @ex8ode and @ex8bc to compute the problem in MATLAB.
4. Achieving the dimensionless form of shear skin relation, Nusselt and Sherwood relations and using them to obtain numeric results.
5. Finally, coding the whole problem in MATLAB and obtaining graphical and numeric outcomes and providing analysis of results.

These equalities are converted to first-order linear equalities with the help of the following newly defined variables. Asymptotic behavior is gained by setting the solution tolerance rate up to $10^{-6}$ and using appropriate initial guesses:

(29) $$f=y_{1},\;\;f^{\prime }=y_{2},\;\;f^{\prime \prime }=y_{3},\;\;f^{\prime \prime \prime }=y_{4},\;\;f^{iv}=y_{4}{}^{\prime }\;g=y_{5},\;\;g^{\prime }=y_{6},\;\;g^{\prime \prime }=y_{6}{}^{\prime },\;\;\theta =y_{7},\;\;\theta^{\prime }=y_{8},\;\;\theta^{\prime \prime }=y_{8}{}^{\prime },\;\;\phi =y_{9},\;\;\phi^{\prime }=y_{10},\;\;\phi^{\prime \prime }=y_{10}{}^{\prime },$$

The Equations $y_{4}{}^{\prime },\;y_{6}{}^{\prime },\;y_{8}{}^{\prime }$ and $y_{10}{}^{\prime }$ takes the following form:

(30) $$y_{4}{}^{\prime }=\left(\left(R*D_{1}*(y_{2}*y_{3}-y_{1}*y_{4})\right)+\left(\left(2*K_{r}*D_{1}*y_{6}\right)\right)+\left(\left(M*D_{4}*y_{3}\right)\right)+\left(Z*y_{3}\right)\;-\left(\gamma *y_{8}*\left(\frac{H_{5}}{H_{1}}\right)\right)\right)*\left({\left(1-\varphi_{1}\right)}^{2.5}*{\left(1-\varphi_{2}\right)}^{2.5}\right)$$

(31) $$y_{6}{}^{\prime }=\left(\left(R*D_{1}*(y_{2}*y_{5}-y_{6}*y_{1})\right)-\left(\left(2*K_{r}*D_{1}*y_{2}\right)\right)+\left(\left(M*D_{4}*y_{5}\right)\right)\;+\left(Z*y_{5}\right)\right)*\left({\left(1-\varphi_{1}\right)}^{2.5}*{\left(1-\varphi_{2}\right)}^{2.5}\right)$$

(32) $$y_{8}{}^{\prime }=\frac{\left(\left(-Pr*R*y_{1}*y_{8}\right)-\left(y_{10}*y_{8}*N_{b}\right)-\left(N_{t}*y_{8}{}^{2}\right)*\left(Q*\frac{y_{6}}{H_{3}}\right)\right)}{\left(1+\frac{1}{D_{3}}\frac{4}{3\;}Rd\right)}$$

(33) $$y_{10}{}^{\prime }=\left(-R*Sc*y_{1}*y_{10}\right)-\left(\frac{Nt}{Nb}\right)*y_{8}{}^{\prime }-\left(\frac{H_{6}}{H_{3}}*K\right)$$

The boundary condition is in the following form:

(34) $$\begin{array}{c} y_{1}=0,\;\;y_{2}=1,\;\;y_{5}=1,\;\;y_{7}=1,\;\;y_{9}=1,\;\;at\;\eta =0\;\;\\ \;\;\;y_{1}=\lambda ,\;\;y_{2}=0,\;\;y_{5}=0,\;\;y_{7}=0,\;\;y_{9}=0,\;\;\;\;\;\;at\;\eta =1\;\; \end{array}\}$$

The skin frictions, Nusselt and Sherwood numbers take the form

(35) $$\begin{array}{c} \tilde{Cf_{x}}=\left(\frac{1}{{\left(1-\varphi_{1}\right)}^{2.5}*{\left(1-\varphi_{2}\right)}^{2.5}}\right)*y_{3},\\ \tilde{Cf_{z}}=\left(\frac{1}{{\left(1-\varphi_{1}\right)}^{2.5}*{\left(1-\varphi_{2}\right)}^{2.5}}\right)*y_{6},\\ Nu_{x}=-\left(H_{3}+\frac{4}{3}Rd\right)*y_{8},\\ Sh_{x}=-y_{10}. \end{array}\}$$

The obtained results show the impact on different profiles. The solution is compared with the literature in Table 1. Thermophysical relations and properties are presented in the following Table 2 and Table 3, respectively.

## 4. Results and Discussion

The results are obtained for different constraints like magnetic field M, porosity Z, heat source/sink *Q*, chemical reaction *Kc*, etc. The reduced skin friction coefficient, Nusselt number coefficient and Sherwood number coefficient are noted for different values of constraints and presented through graphs and tables in the following.

### 4.1. Velocity Profiles

**Figure 2:**
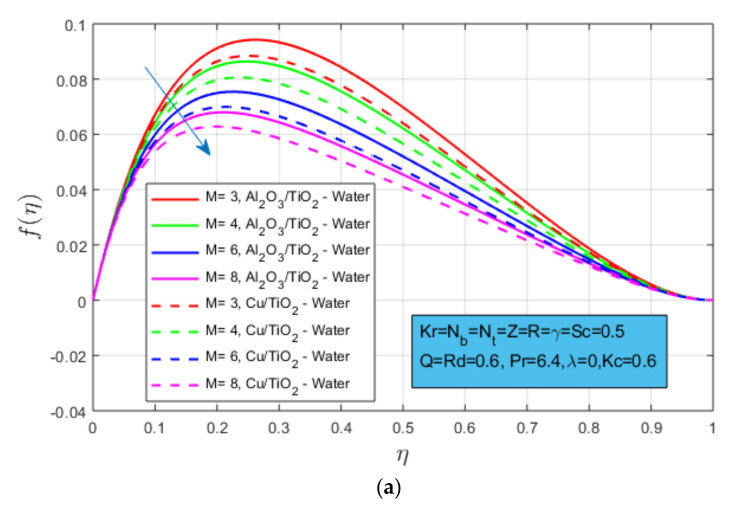

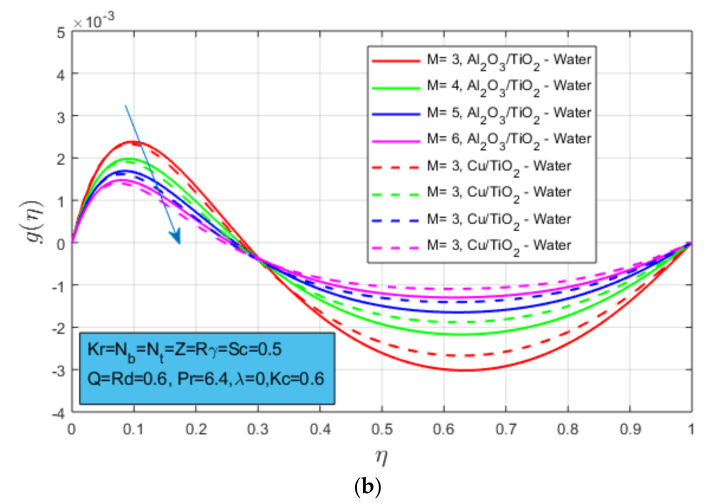

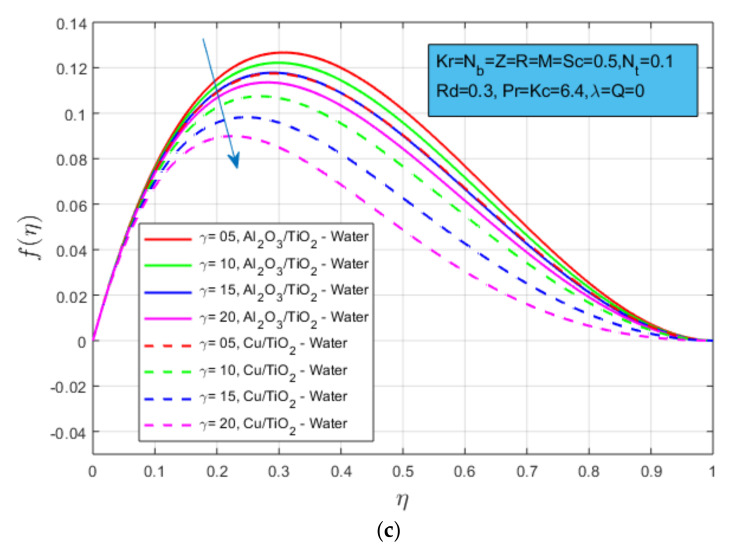

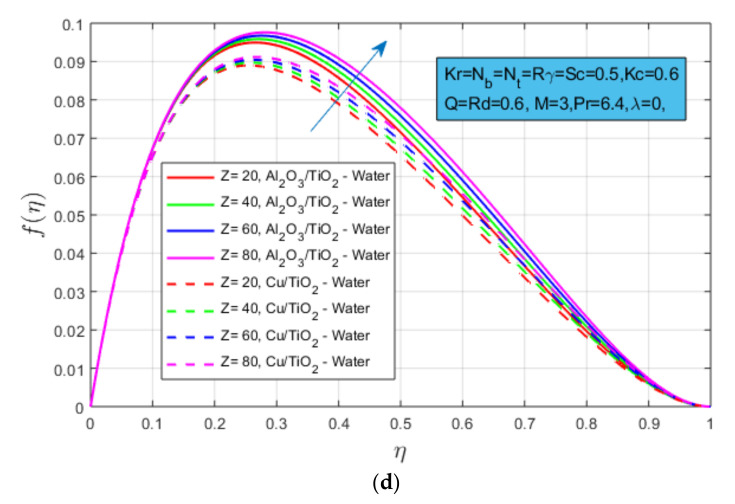

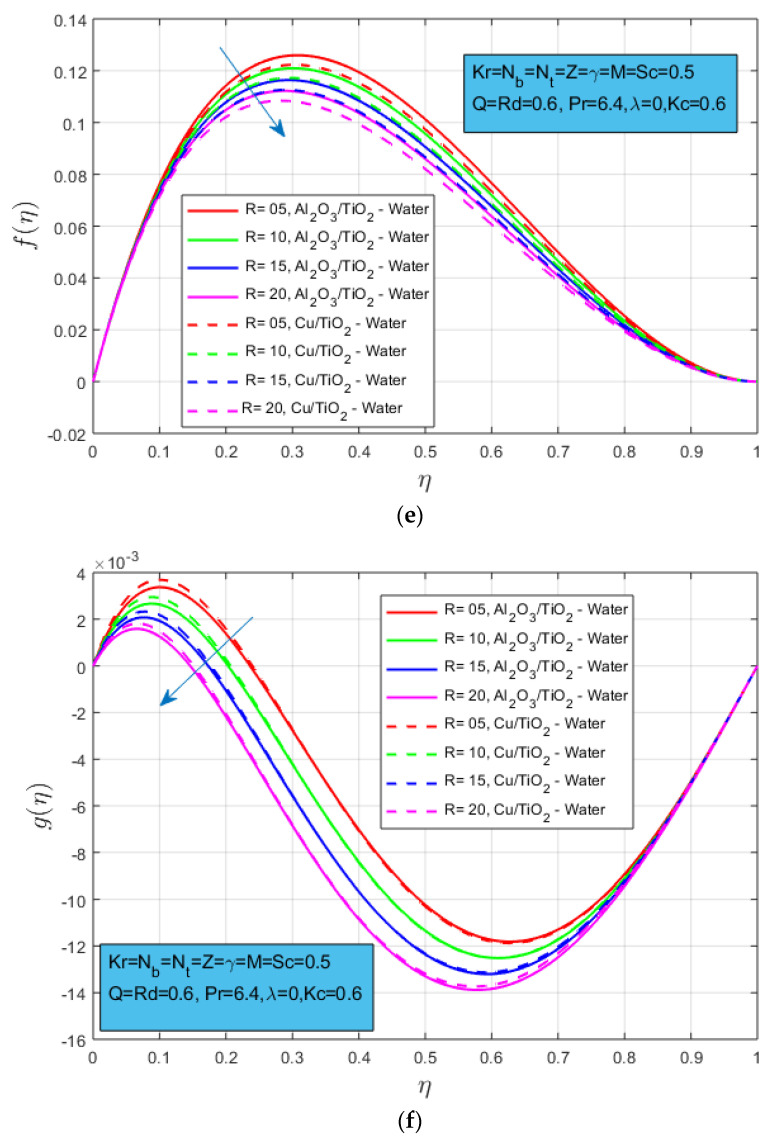

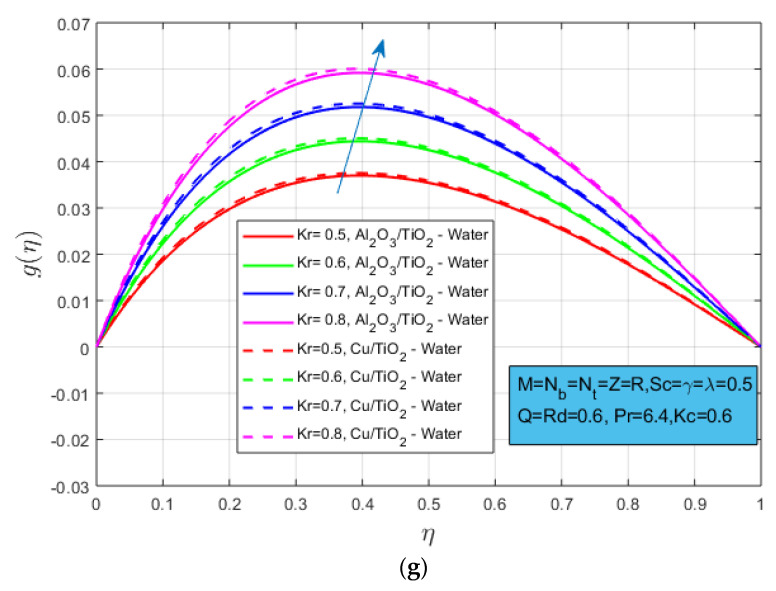
(**a**) Influence of magnetic field *M* on velocity constitute f(η); (**b**) influence of magnetic field *M* on velocity constitute g(η); (**c**) influence of mixed convection constraint *γ* on velocity constitute f(η); (**d**) influence of rotation constraint Kr on velocity constitute f(η); (**e**) influence of reynolds number *R* on velocity constitute f(η); (**f**) influence of reynolds number *R* on velocity constitute g(η); and (**g**) influence of rotation parameter Kr on velocity constitute g(η).

The impact of magnetic field M is presented in Figure 2a,b for both types of hybrid nanofluid, i.e., $Al_{2}O_{3}/TiO_{2}-water$ and $CuO/TiO_{2}-water$ (say oxide particles hybrid nanofluid and mixed particles hybrid nanofluid, respectively, throughout the study). It is observed that both velocity profiles f(η) and g(η) decays by increasing the magnetic field strength. This phenomenon occurs due to generated resistive force in the vicinity of the boundary layer known as Lorentz force. It provides resistance to fluid form flowing smoothly. Whereas clear differences in the magnitude of distinct velocity profiles can be observed. Oxide nanoparticles hybrid nanofluid have higher magnitudes as compared to the mixed nanoparticles hybrid nanofluid. The reason is the low density and higher density of nanoparticles respectively. Additionally, the momentum boundary layer of both examined hybrid nanofluids for primary and secondary velocities have increased with an increment in magnetization force.

Figure 2c presents the thermal slip γ on velocity profile f(η). As the thermal slip parameter increases, the velocity profile decreases and a sharp slowdown is observed in the case of mixed nanoparticles hybrid nanofluid as compared to oxide nanoparticles hybrid nanofluid. Due to the combination of the dense particle, the velocity decays rapidly. The influence of the porosity parameter Z is shown in Figure 2d. Their direct relation is noted for the porosity and velocity profile for both hybrid nanofluids. As the permeability of the medium increases, the velocity profile also increases, and higher/greater velocity is observed in the case of the oxide nanoparticles hybrid nanofluid. Fluid flow and Reynold number R have an inverse relation, which is shown in Figure 2e,f for velocity profile f(η). and g(η), respectively. The effect of rotation parameter Kr on velocity profile g(η) is shown in Figure 2g. The velocity profile g(η) for both hybrid nanofluid increases by rising rotation parameter and higher velocity is noted for mixed nanoparticles hybrid nanofluid.

### 4.2. Temperature Profile

**Figure 3:**
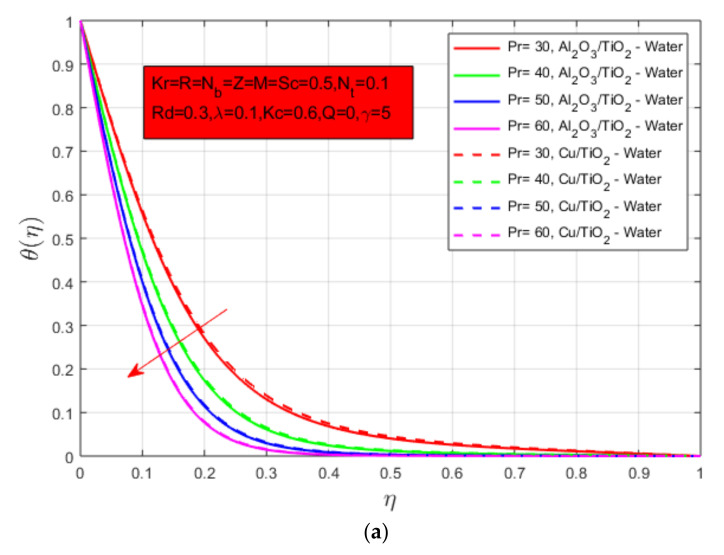

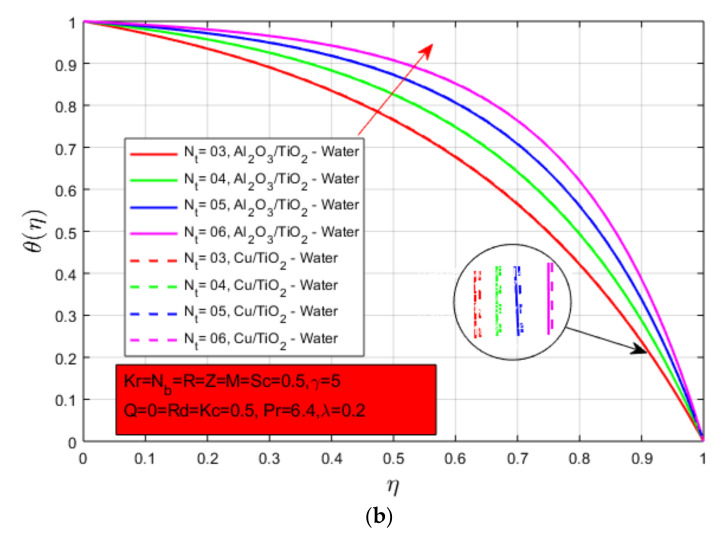

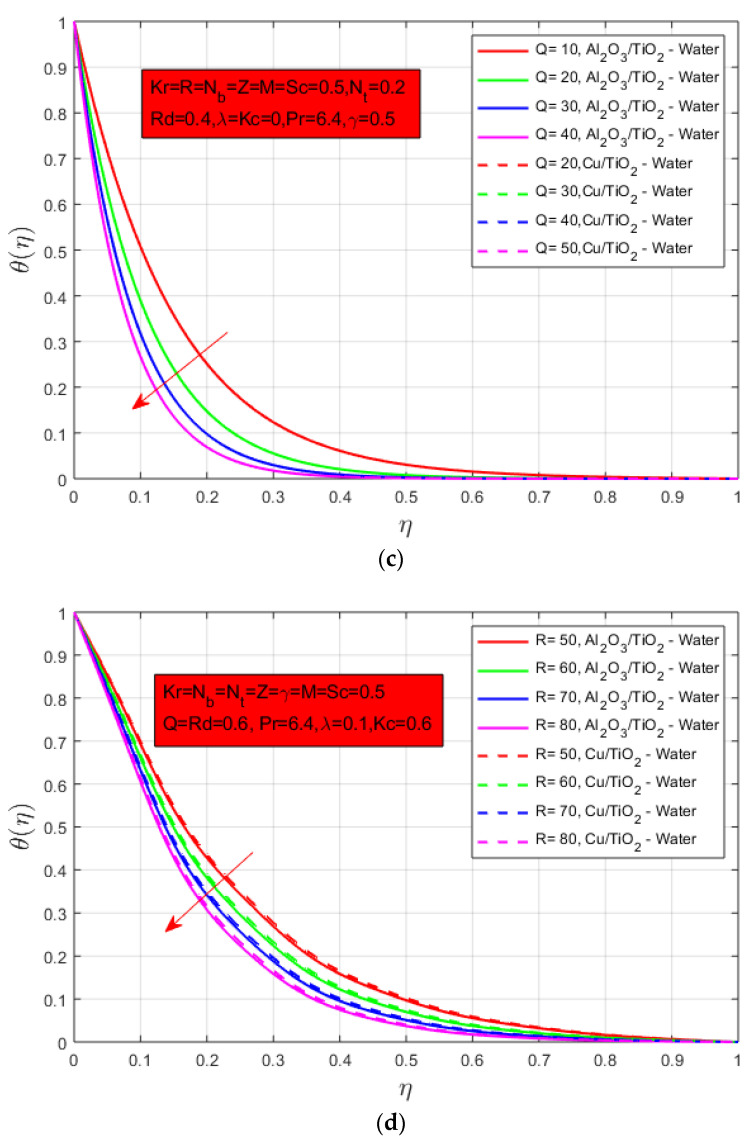
(**a**) Influence of Prandtl number Pr on temperature constitute θ(η); (**b**) influence of thermophoresis parameter $N_{t}$ on temperature constitute θ(η); (**c**) influence of heat source/sink constraint Q on temperature constitute θ(η); and (**d**) influence of Reynold number *R* on temperature constitute θ(η).

The influence on the temperature profile of Prandtl number Pr is shown in Figure 3a for both hybrid nanofluids. Prandtl number and thermal diffusivity have inverse relations between them, so the temperature profile decays with an increase in the Prandtl number of both hybrid nanofluids. Oxide nanoparticles have a lower density as compared to mixed hybrid nanoparticles, so they face a rapid rise and fall in the temperature boundary layer. The impact of thermophoresis parameter $N_{t}$ on temperature profile θ(η) is presented in Figure 3b. Thermophoresis is defined as the phenomenon in which moving particles exhibit different behavior to the force of temperature gradient. Movement of light molecules to a hot region and vice versa balance the temperature of the fluid. The thermophoresis parameter $N_{t}$ and temperature profile have a direct relation, so the temperature profile increases when the thermophoresis parameter $N_{t}$ increases. Heat source/sink parameter Q has a similar influence on the temperature profile as shown in Figure 3c for both hybrid nanofluids. The thermal boundary layer decays by a rise in heat source/sink parameter Q. The impact of Reynold number R on the temperature profile is shown in Figure 3d. As the Reynold number increases, the temperature profile decreases, and a lower thermal boundary layer was observed for oxide nanoparticles hybrid nanofluid.

### 4.3. Concentration Profile

**Figure 4:**
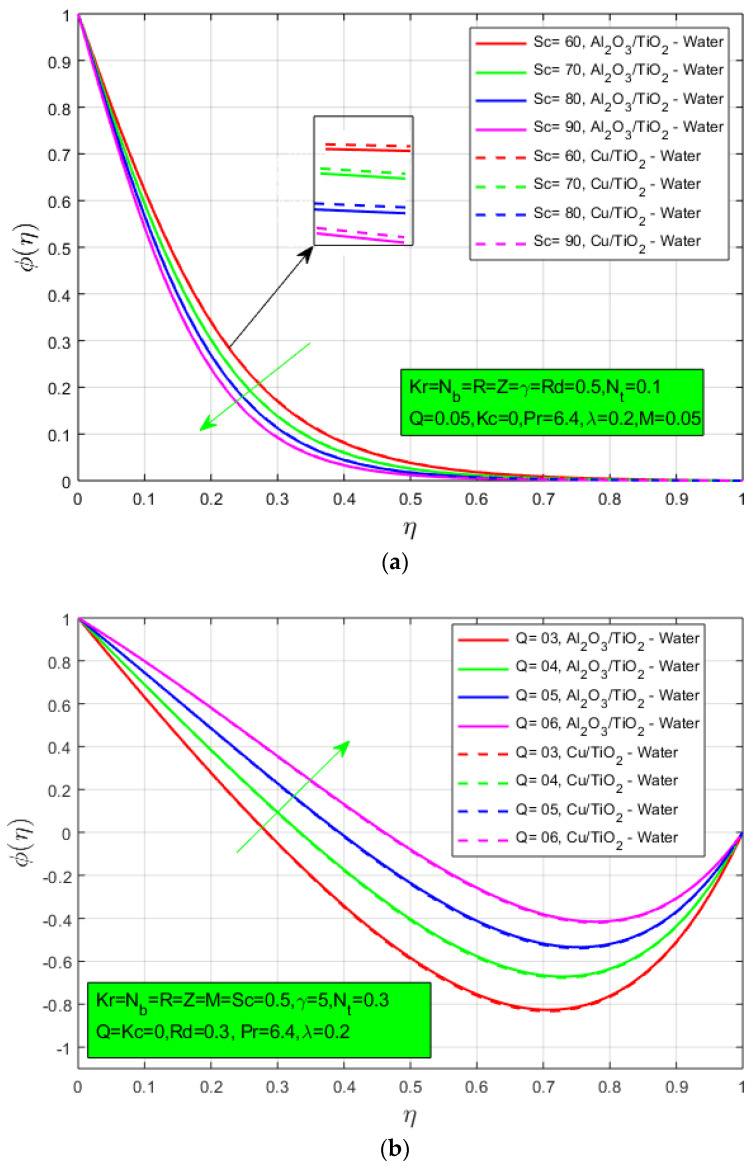

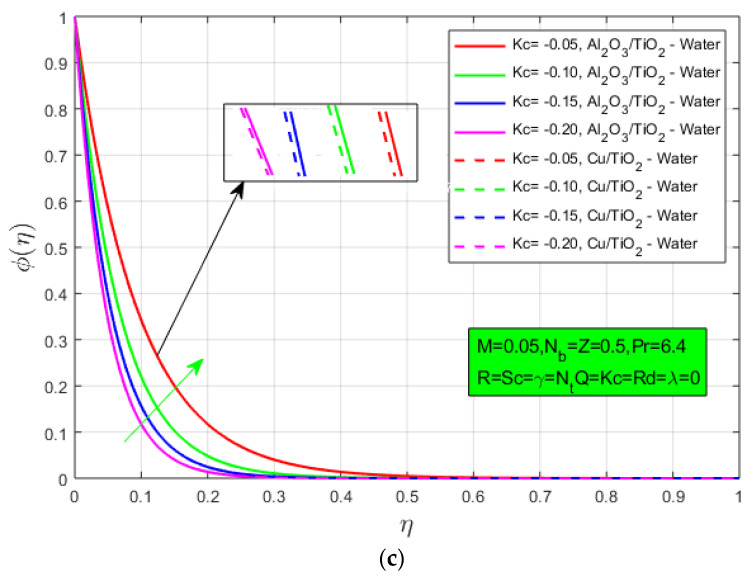
(**a**) Influence of Schmidt number Sc, on concentration constitute ϕ(η); (**b**) influence of heat source/sink constraint *Q* on concentration constitute ϕ(η); and (**c**) influence of chemical reaction Kc on concentration constitute ϕ(η).

The results obtained for Schmidt number Sc, heat source/sink Q and chemical response Kc on concentration profile ϕ are shown in the following figures. Figure 4a shows the effect of Schmidt number on concentration profile ϕ. It is a relation of momentum diffusivity and mass diffusivity. The concentration profile decays when the Schmidt number increases and a lower concentration boundary layer is noted for mixed nanoparticles hybrid nanofluid. The heat source/sink parameter Q has a direct relation with the concentration profile. The concentration profile ϕ increases when the heat source/sink increases as shown in Figure 4b. The impact of chemical response Kc for the concentration profile is presented in Figure 4c. The concentration boundary layer expands by increasing the chemical reaction parameter Kc because it has a direct relation with the chemical reaction parameter Kc. The higher concentration boundary layer is noticed for oxide nanoparticles hybrid nanofluid.

### 4.4. Skin Frictions, Nusselt and Sherwood Numbers

The numerical outcomes for various study parameters are presented in the form of the tables given below. Table 4 indicates the influence of different values of the rotation parameter, Reynolds number, magnetic field parameter, mixed convection parameter and permeability parameter on skin frictions $\tilde{\;Cf_{x}}$ and $\tilde{\;Cf_{z}}$ for both types of hybrid nanofluid. The rotation parameter Kr . increases the skin friction for both oxide nanoparticles hybrid nanofluid and mixed nanoparticles hybrid nanofluids. The internal movement of fluid particles caused by various fluid velocities is measured by the Reynolds number. The Reynolds number decreases the skin friction along the x-direction, while it increases along the *z*-axis. Similar behavior is noted for magnetic field parameter M. When the mixed convection and permeability parameter increases, an opposite behavior to the rotation parameter is observed for both hybrid nanofluids. Table 5 shows the influence of the Reynolds number, permeability parameter, chemical reaction parameter, rotation parameter magnetic parameter mixed convection parameter on the Nusselt number $Nu_{x}$ and $Sh_{x}\;$ the number for both oxide nanoparticles and mixed nanoparticles hybrid nanofluid. The Nusselt and Sherwood number increase for the rise in Reynolds number, permeability parameter and chemical reaction parameter while it decays for the rise in rotation parameter, magnetic parameter, and mixed convection parameter. Similarly, Table 6 shows the thermophoresis, Schmidt number, Prandtl number, radiation parameter and heat source/sink parameter on Nusselt and Sherwood numbers. The heat and mass transfer rate increases for an increase in Prandtl number, radiation parameter and heat source/sink parameter while it decreases for thermophoresis and Schmidt number.

## 5. Conclusions

This research article investigates the 3D mixed convection oxide nanoparticles hybrid nanofluid and mixed nanoparticles hybrid nanofluid flow in a permeable rotating system under the influence of a magnetic field and heat source/sink. The governing equationsare transformed into ordinary differential equations by using similarity transformations. The boundary value problem technique is used to tackle the transformed governing equations at MATLAB by setting the solution tolerance rate 10^−6^. Graphs and tables are used to present the obtained outcomes. The major outcomes of this comparative research are listed below for velocity, concentration, temperature, Nusselt number and Sherwood number.

1. The increase in the magnetic field parameter increases the resistance to flow so the velocity profile decays with an increase in the magnetic parameter.
2. The porosity and rotation parameter increases the velocity profile while the Reynolds number and mixed convection parameter decay.
3. Thermophoresis parameters have a direct relation with the temperature profile, whereas heat source/sink, Prandtl number and Reynolds number have an inverse relation with the temperature profile.
4. Heat source/sink does not have a prominent effect on the concentration profile.
5. Schmidt number and chemical reaction parameter decay the concentration while the heat source/sink parameter increases.
6. Skin friction along the *x*-axis decreases and the *z*-axis increases by increasing the Reynolds number for both hybrid nanofluids.
7. Reduced skin friction and high Nusselt number are being observed for oxide nanoparticles hybrid nanofluid.

## Figures and Tables

**Table 1:** The comparison of current outcomes with the literature.

λ	Present Outcomes for Kr			Sheikholeslami and Ganji Kr [46]		
	0.5	2	4	0.5	2	4
1	2.633501	2.633502	2.633515	2.63350	2.63350	2.63351
2	3.271112	3.271754	3.274182	3.27111	3.27175	3.27418
3	3.745803	3.746081	3.747421	3.74580	3.74607	3.74742

**Table 2:** Thermophysical relations of nanoparticles and base fluid.

Properties	Hybrid Nanofluid
Density	$\rho_{hnf}=\left(1-(\phi_{1}+\phi_{2}\right))\rho_{f}+\phi_{1}\rho_{s1}+\phi_{2}\rho_{s2}$
Dynamic Viscosity	$\mu_{hnf}=\frac{\mu_{f}}{{\left[1-\left(\phi_{1}+\phi_{2}\right)\right]}^{5/2}}$
Heat Capacity	${\left(\rho C_{p}\right)}_{hnf}=\left[1-\left(\phi_{1}+\phi_{2}\right)\right]{\left(\rho c_{p}\right)}_{f}+\phi_{1}{\left(\rho c_{p}\right)}_{s1}+\phi_{2}{\left(\rho c_{p}\right)}_{s2}$
Thermal Conductivity	$\frac{k_{hnf}}{k_{f}}=\frac{\left(k_{s1}+k_{s2}\right)+2k_{f}\left(1-\left(\phi_{1}+\phi_{2}\right)\right)+2\phi_{1}k_{s1}+2\phi_{2}k_{s2}}{\left(k_{s1}+k_{s2}\right)+\left(2+\left(\phi_{1}+\phi_{2}\right)\right)k_{f}-\left(\phi_{1}k_{s1}+\phi_{2}k_{s2}\right)}$
Electrical Conductivity	$\frac{\sigma_{hnf}}{\sigma_{f}}=1+\frac{3\left[\frac{\sigma s_{1}\;\phi_{1}+\sigma_{s2}\;\phi_{2}}{\sigma_{f}}-\left(\phi_{1}+\phi_{2}\right)\right]}{\left(2+\frac{\sigma_{s1}+\sigma_{s2}}{\sigma_{f}}\right)-\left[\frac{\sigma_{s1}\;\phi_{1}+\sigma_{s2}\;\phi_{2}}{\sigma_{f}}\right]+\left(\phi_{1}+\phi_{2}\right)}$

**Table 3:** Thermophysical properties of nanoparticles and base fluid.

Properties	*ρ* (kg/m^3^)	*Cp* (J/kg K)	*K* (W/m K)	*σ* (Ω·m)^−1^	$\beta {\left(K\right)}^{-1}\;$
Water	997.1	4179	0.613	5 × 10^−2^	21 × 10^−5^
C*u*	8933	385	400	5.96 × 10^7^	1.67 × 10^−5^
$Al_{2}O_{3}$	3970	765	40	1 × 10^−9^	0.85 × 10^−5^
$TiO_{2}$	4250	686.2	8.96	6.27 × 10^−5^	0.9 × 10^−5^

**Table 4:** The reduced skin frictions $\tilde{\;Cf_{x}}$ and$\tilde{\;Cf_{z}}$ for $Al_{2}O_{3}/TiO_{2}$*-water* and $Cu/TiO_{2}$*-water* hybrid nanofluid when Pr=6.3, Nt=Q=Kc= Rd =Sc=0.5.

R	Kr	M	Z	γ	$\tilde{Cf_{x}}\;$ $\left(Cu/TiO_{2}-\mathrm{Water}\right)$	$\tilde{Cf_{x}}\;$ $(Al_{2}O_{3}/TiO_{2}$ -Water)	$\tilde{Cf_{z}}$ $(Cu/TiO_{2}$ -Water)	$\tilde{Cf_{z}}\;$ $(Al_{2}O_{3}/TiO_{2}$ -Water)
0.5	0.5	0.5	0.5	0.5	−2.11328	−1.93874	0.291381	0.277788
0.6					−2.11820	−1.94371	0.290821	0.277224
0.7					−2.12311	−1.94866	0.290251	0.276662
0.8					−2.12801	−1.95360	0.289694	0.276103
0.5	02	0.5	0.5	0.5	−2.08725	−1.91522	1.16450	1.11024
	04				−2.00328	−1.83948	2.32261	2.21487
	06				−1.86147	−1.71183	3.46923	3.30924
	08				−1.66003	−1.53085	4.60146	4.39035
0.5	0.5	0.2	0.5	0.5	−1.66849	−1.56202	0.365014	0.339169
		0.4			−1.97204	−1.81824	0.311316	0.294825
		0.6			−2.24845	−2.05475	0.274541	0.263144
		0.8			−2.50282	−2.27474	0.24751	0.239182
0.5	0.5	0.5	05	0.5	−2.30332	−2.13388	0.330034	0.320293
			06		−2.34519	−2.17685	0.34121	0.332983
			07		−2.38690	−2.21966	0.353697.	0.347387
			08		−2.42847	−2.26231	0.36775	0.36389
0.5	0.5	0.5	0.5	0.6	−2.12701	−1.94437	0.291221	0.277724
				0.7	−2.14073	−1.95011	0.291056	0.277661
				0.8	−2.15446	−1.95562	0.290891	0.277598
				0.9	−2.16819	−1.96125	0.290725	0.277535

**Table 5:** Outcomes of Nusselt and Sherwood number $Nu_{x}$ and$\;Sh_{x}$ for ${\mathrm{Al}}_{2}O_{3}/{\mathrm{TiO}}_{2}$-water and $Cu/TiO_{2}$*-water* hybrid nanofluid when Pr=6.3, Nt=Q=Rd=Sc=0.5.

R	Kr	M	Z	γ	Kc	$Nu_{x}\;$ $(Cu/TiO_{2}$ -Water)	$Nu_{x}$ $(Al_{2}O_{3}/TiO_{2}$ -Water)	$Sh_{x}\;$ $(Cu/TiO_{2}$ -Water)	$Sh_{x}$ $(Al_{2}O_{3}/TiO_{2}$ -Water)
0.5	0.5	0.5	0.5	0.5	0.5	1.67292	1.68783	−50.8965	−45.0512
0.6						1.71408	1.72957	−50.8515	−45.0124
0.7						1.75548	1.77157	−50.8063	−44.9735
0.8						1.79711	1.81378	−50.7609	−44.9344
0.5	02	0.5	0.5	0.5	0.5	1.6727	1.68763	−50.8967	−45.0513
	04					1.67202	1.68702	−50.8972	−45.0517
	06					1.67098	1.68607	−50.8980	−45.0523
	08					1.66969	1.68488	−50.8991	−45.0532
0.5	0.5	0.2	0.5	0.5	0.5	1.67782	1.69208	−50.8937	−45.049
		0.4				1.67444	1.68916	−50.8956	−45.0505
		0.6				1.67151	1.68657	−50.8974	−45.0518
		0.8				1.66894	1.68426	−50.8989	−45.053
0.5	0.5	0.5	05	0.5	05	1.671	1.68583	−50.8976	−45.0522
			06			1.67057	1.68539	−50.8979	−45.0524
			07			1.67015	1.68494	−50.8981	−45.0526
			08			1.66972	1.6845	−50.8984	−45.0528
0.5	0.5	0.5	0.5	0.6	0.5	1.67279	1.68777	−50.8966	−45.0512
				0.7		1.67266	1.68772	−50.8967	−45.0512
				0.8		1.67253	1.68766	−50.8968	−45.0512
				0.9		1.67239	1.68761	−50.8968	−45.0513
0.5	0.5	0.5	0.5	0.5	0.2	1.59652	1.62653	−70.1406	−60.6065
					0.3	1.67905	1.69262	−53.0924	−46.205
					0.4	1.69542	1.69995	−26.6378	−22.5092
					0.5	1.71541	1.71557	−0.885862	2.5687

**Table 6:** Outcomes of Nusselt and Sherwood number $Nu_{x}$ and$\;Sh_{x}$ for $Al_{2}O_{3}/TiO_{2}$*-water* and $Cu/TiO_{2}$*-water* hybrid nanofluid when R=Kr=M=Z=0.5.

Nt	Pr	Rd	Sc	Q	$Nu_{x}\;$ $(Cu/TiO_{2}$ -Water)	$Nu_{x}$ $(Al_{2}O_{3}/TiO_{2}$ -Water)	$Sh_{x}\;$ $(Cu/TiO_{2}$ -Water)	$Sh_{x}$ $(Al_{2}O_{3}/TiO_{2}$ -Water)
0.5	6.3	0.5	0.5	0.5	1.67614	1.6911	−50.8951	−45.0499
0.6					1.6262	1.64022	−50.9590	−45.1011
0.7					1.57829	1.59145	−51.0421	−45.1688
0.8					1.53233	1.54471	−51.144	−45.2526
0.5	10	0.5	0.5	0.5	1.79301	1.80991	−50.8421	−45.0038
	15				1.95798	1.97754	−50.7675	−44.9381
	20				2.12475	2.14689	−50.6937	−44.8726
	25				2.29210	2.3167	−50.6222	−44.8086
0.5	6.3	0.3	0.5	0.5	1.84330	1.86321	−50.9273	−45.0761
		0.4			1.89737	1.9159	−50.9104	−45.0623
		0.6			1.9911	2.00716	−50.8851	−45.0421
		0.8			2.06923	2.08319	−50.8679	−45.0286
0.5	6.3	0.5	05	0.5	1.74568	1.74682	−22.6591	−19.7974
			06		1.69653	1.70747	−49.0909	−43.5017
			07		1.69988	1.71023	−48.694	−43.1596
			08		1.70299	1.71279	−48.2778	−42.8004
0.5	6.3	0.5	0.5	0.1	1.57487	1.59186	−50.8721	−45.0312
				0.2	1.59961	1.61607	−50.8782	−45.0362
				0.3	1.62419	1.64013	−50.8843	−45.0412
				0.4	1.64863	1.66405	−50.8904	−45.0462

## Data Availability

All data relevant to the research is included in the manuscript.

## Outcomes and Future Implications

In this research, two different hybrid nanofluid flows are discussed with different assumptions. The major outcomes are found to have a high Nusselt number and reduced skin friction for oxide nanoparticles hybrid nanofluids. Such hybrid nanofluids can be used in chemical engineering, electronic devices, etc., and in the future, advances in such types of research can be achieved to enhance the performance.

## Nomenclature

$p^{*}({\mathrm{Kgm}}^{-1\;}s^{-2})$	Modified fluid pressure
$k({\mathrm{Wm}}^{-1\;}K^{-1})$	Thermal conductivity
$B_{0}\left(A/m\right)$	Constant magnetic field
$Sh_{x}$	Sherwood number
*Kc* (mol/s)	Chemical reaction parameter
γ	Mixed convection parameter
$H,H_{1},\;H_{2},H_{3},\;H_{4},H_{5},H_{6}$	Constants
u,v,*w* (m$s^{-1}$*)*	Velocity components in x,y,z direction
*Z*	Porosity parameter
$f,g\left({\mathrm{ms}}^{-1}\right)$	Dimensionless velocity,
h(m)	Distance between plates
$N_{b},N_{t}$	Brownian diffusion and Thermophoresis parameter
$v_{f}\left(m^{2}s^{-1}\right)$	Kinematic viscosity
$\mu_{f}\left({\mathrm{Nsm}}^{-2}\right)$	Dynamic viscosity
*3D*	Three dimensional
$K_{r}$	Rotation parameter
$C_{p}\left({\mathrm{Jkg}}^{-1\;}K^{-1}\right)$	Specific heat at constant pressure
M	Magnetic parameter
$D_{B},D_{T}$	Brownian and Thermophoresis diffusion
*Q* (J)	Heat source/sink parameter
$Nu_{x}$	Nusselt number
η	Similarity variable
T(K), $C\left(\mathrm{mol}/m^{3}\right)$	Temperature and concentration
$\tilde{Cf},\;Cf$	Skin friction coefficients
a(m)	Stretching rate
Pr,Ec	Prandtl and Eckert number
Rd	Thermal radiation parameter
$q_{w}\left({\mathrm{Wm}}^{-2}\right),$	Heat flux
$q_{m}\left({\mathrm{kgm}}^{-2\;}s^{-1}\right)$	Mass flux
**Greek symbols**	
$\tau_{w}\left({\mathrm{Nm}}^{-2}\right)$	Shear stress
$\sigma \left({\Omega m}^{-1}\right)$	Electrical conductivity
θ,ϕ	Dimensionless temperature and concentration
$\Omega \left({\mathrm{ms}}^{-1}\right)$	Rotational velocity
$\rho \left({\mathrm{kgm}}^{-3}\right)$	Density
$\alpha \left(m^{-2\;}s^{-1}\right)$	Temperature diffusivity
